# Supplementary material for: Genomic diversity of wild and cultured Yesso scallop Mizuhopecten yessoensis from Japan and Canada
Source: G3 (Bethesda). 2023 Oct 19;13(12):jkad242. doi: 10.1093/g3journal/jkad242 (PMC10700054; doi:10.1093/g3journal/jkad242)
Supplement: jkad242_Supplementary_Data [file jkad242_supplementary_data.zip › File_S6_G3-2023-404594.pdf]

Additional File S6. Principal components analysis (PCA; A, B) and population averaged F_{ST} (showing 95% confidence intervals; C) on the dataset with putative close relatives still included. Both results use single-SNP per locus data.

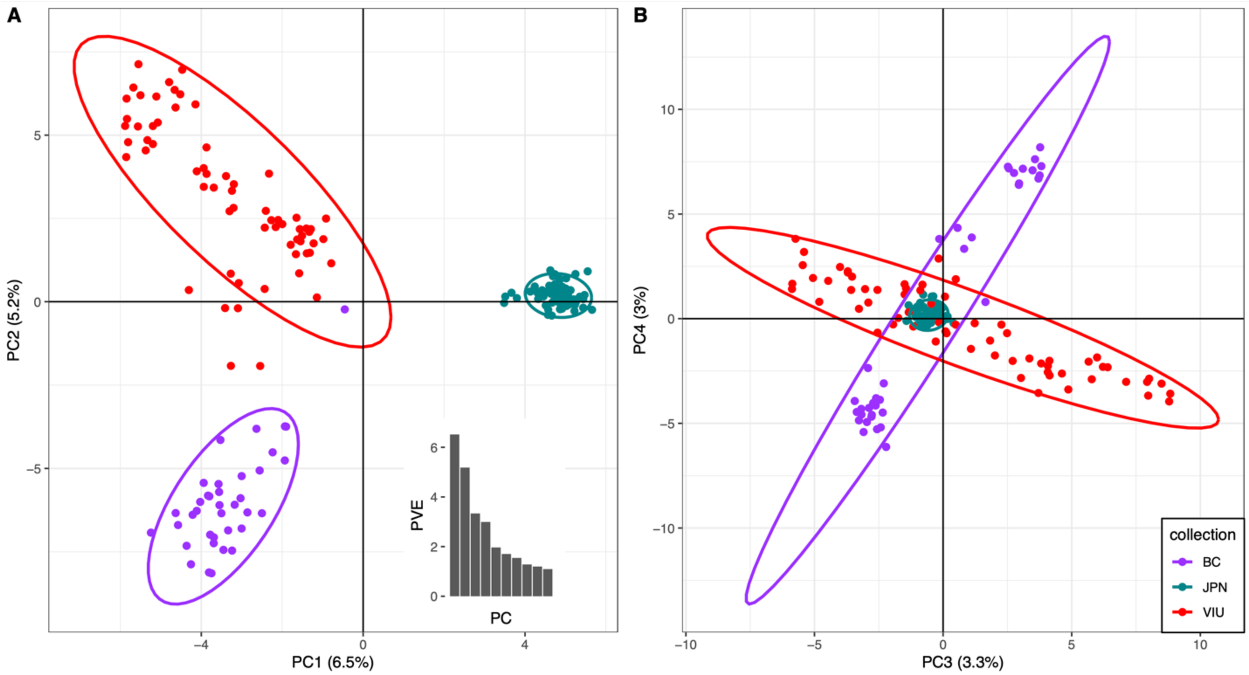

C

	**BC**	**JPN**	**VIU**
**BC**	-	0.101	0.085
**JPN**	0.093	-	0.074
**VIU**	0.076	0.067	-
