# Supplementary material for: Genomic diversity of wild and cultured Yesso scallop Mizuhopecten yessoensis from Japan and Canada
Source: G3 (Bethesda). 2023 Oct 19;13(12):jkad242. doi: 10.1093/g3journal/jkad242 (PMC10700054; doi:10.1093/g3journal/jkad242)
Supplement: jkad242_Supplementary_Data [file jkad242_supplementary_data.zip › Supplemental_Material_Legends_G3-2023-404594.docx]

File S1. Sample metadata including sources and dates of collections.

File S2. Per sample number of reads, alignments, and alignment rate.

File S3. Number of variants and alleles per RAD-tag, including summary tables.

File S4. Effective population size (*N_e_*) results using microhaplotypes or single variant per tag.

File S5. Per locus stats including *F*_ST_, *H*_OBS_ (global) for each locus in the filtered, single-SNP per locus dataset, H_OBS_ in each population, and Hardy-Weinberg equilibrium test outputs for single SNPs or microhaplotypes per population. Per locus stats after putative close relative removal also included.

File S6. PCA and FST analysis prior to putative close relative removal.
